# Endovascular recanalization of symptomatic chronic cerebral artery occlusion: predictors for successful recanalization and perioperative complications

**DOI:** 10.3389/fneur.2025.1453841

**Published:** 2025-04-11

**Authors:** Xueqian Zhang, Yang Li, Kuochang Yin, Zhiwei Hao, Yidian Fu, Qishuo Yang, Guodong Xu, Peiyuan Lv

**Affiliations:** ^1^Department of Neurology, Hebei Medical University, Shijiazhuang, China; ^2^Department of Neurology, Hebei General Hospital, Shijiazhuang, China; ^3^Hebei Provincial Key Laboratory of Cerebral Networks and Cognitive Disorders, Shijiazhuang, China; ^4^Department of Staff Hospital, Hebei Normal University, Shijiazhuang, China; ^5^Department of Neurology, Graduate School of Hebei North University, Zhangjiakou, China

**Keywords:** chronic cerebral artery occlusion, endovascular treatment, chronically occluded internal carotid artery, basilar artery occlusion, neutrophil-to-lymphocyte ratio

## Abstract

**Background and purpose:**

Endovascular recanalization and stenting has been used to treat patients with symptomatic chronic cerebral artery occlusion, including intracranial vertebrobasilar artery occlusion and internal carotid artery occlusion. Our challenge is to improve success rates and reduce the incidence of postoperative complications. This study sought to identify potential predictors for successful recanalization.

**Methods:**

Our study included 103 consecutive patients between February 2021 and October 2024 with symptomatic chronic cerebral artery occlusion who were treated with endovascular recanalization. We recorded clinical information, laboratory and examination results, radiologic characteristics and procedural results of patients. Factors affecting surgical outcomes were analyzed by univariate and multivariate analyses.

**Results:**

A total of 103 consecutive CCAO recanalization attempts were performed from February 2021 to October 2024 in 103 patients (78 men; age 61.1 ± 11.1 years; range: 32–81 years) with overall technical success rate 68.9%. Patients had chronic comorbidities such as hypertension (78, 75.7%), diabetes mellitus (32, 31.10%), and cardiac disease (12, 11.7%). 38 (36.9%) had a history of smoking, and 23 (22.3%) had a history of drinking. The rate of overall intraoperative complication was 10.7% (11/103). Multivariate analysis showed that stump morphology, smoking history, duration from last neurologic event (longer than 6 months or not), age, NLR were significantly associated with successful recanalization. According to the coefficients of the prediction model, the technical success rates were 100, 66.7 and 11.1% in patients with ≤6, 6–10, ≥10 points, respectively.

**Conclusion:**

The morphology of occluded stumps, duration from last neurologic event, age, smoking history and NLR can be used to predict the outcome of vascular recanalization.

## Highlights

- This study covers a wide range of cases including internal carotid artery and vertebrobasilar artery system.
- This study identified five factors that affect the outcome of vascular recanalization.
- This study developed a scoring system for predicting surgical outcomes.

## Introduction

Chronic cerebral artery occlusion disease is currently the culprit that troubles a considerable number of cerebrovascular diseases patients with high recurrence rate and low quality of life. According to the location of vascular occlusion, it can be divided into anterior circulation occlusion and posterior circulation occlusion. The treatment of patients with chronic cerebral artery occlusion (CCAO) is controversial, especially those with symptoms of hypoperfusion or stroke. Even with active internal medicine medication treatment, 6–20% of CCAO patients still experience stroke events every year. The risk of ischemic stroke in such patients increased from 8% after 30 days of follow-up to 14% after 5 years of follow-up (1). The annual risk of recurrent stroke in patients with transient ischemic attack (TIA) or ischemic stroke associated with carotid artery occlusion is approximately 5–6% (2). That risk is approximately 12% per year in the subgroup of symptomatic ICA occlusive patients, in this subgroup, the hemodynamic state of the brain is impaired (3).

The proportion of patients with posterior circulation occlusion is not low in cerebral artery occlusive diseases. About 20% of ischemic stroke patients are caused by posterior circulation ischemia (4). According to literature statistics, the incidence of chronic vertebral artery occlusion in the Chinese population is higher than that in Caucasian (5). In patients with posterior circulation ischemia, the incidence of significant stenosis or occlusion at the origin of the vertebral artery is 32.1%, and the incidence of significant stenosis or occlusion in the intracranial segment of the vertebral artery is 32.4%; The incidence of vertebral artery stenosis or occlusion is very high in patients with posterior circulation ischemia (6). With the rapid development of neural intervention technology and surgical instruments, intervention recanalization technology has become increasingly mature. The number of research on chronic intracranial and extracranial artery occlusion and recanalization is also increasing year by year both domestically and internationally.

However, almost all studies only focus on anterior circulation occlusion, there is relatively few research on posterior circulation occlusion. In our study, we analyzed and summarized various cases of chronic cerebral artery occlusion treatment completed in the past 5 years, including the intracranial and extracranial segments of anterior circulation, as well as the intracranial and extracranial segments of posterior circulation. In the process, we analyzed and explored the feasibility, safety, and predictive factors for surgical success.

## Methods

### Overall design

This study is a retrospective study conducted at our center. Every patient with CCAO were treated in a unified clinical management process including patient screening and exclusion criteria, surgical process management, perioperative management, and long-term follow-up. All patients have signed written informed consent forms. This study followed the principles of the Declaration of Helsinki and was approved by the Ethics Committee of Hebei General Hospital (No. 2024-LW-084).

### Patient enrollment

All patients had received intensive medical treatment before intravascular treatment (including antiplatelet therapy and controlling atherosclerosis risk factors: blood pressure, plasma glucose, plasma lipid, smoking cessation, alcohol abstinence, etc.), but because of inadequate response to medication, there were still symptoms of cerebral ischemia caused by hypoperfusion. The responsible arteries of the selected patients include complete occlusion of the internal carotid artery (both the intracranial and extracranial segments), the middle cerebral artery, the vertebral artery (both the intracranial and extracranial segments) and the basilar artery. We recorded the time interval from the last appearance of symptoms (including ischemic stroke and transient ischemic attack) to surgery for each patient (each patient was over 30 days).

### The inclusion criteria

1. Age > 18 years;
2. Atherosclerotic CCAO defined as 100% cross-sectional truncation of the vascular lumen documented by computerized magnetic resonance angiography (MRA) or tomography angiography (CTA) and uniformly confirmed with digital subtraction angography (DSA);
3. Time from imaging diagnosis or from aggravation of clinical symptoms defined as change in National Institutes of Health Stroke Scale (NIHSS) score ≥ 4 or modified Rankin Scale (mRS) score ≥ 1 to recanalization was over 24 h;
4. Even after receiving active pharmacological treatment, the recurrence or exacerbation of neurological symptoms could not be ameliorated or controlled;
5. Perfusion imaging (CTP or MRP) shows low perfusion in the supply area of occluded arteries;
6. Each patient has at least one atherosclerosis risk factor such as hypertension, dyslipidemia, diabetes mellitus, smoking, etc.

### The exclusion criteria

1. Nonatherosclerotic occlusion (vasculitis, trauma, dissection, or Moyamoya disease, etc.) or asymptomatic CCAO;
2. Known allergy or contraindication to heparin, aspirin, clopidogrel, metal, or general anesthesia;
3. Expected lifespan is less than 2 years caused by any other medical conditions;
4. Recurrence or aggravation of neurologic symptoms are not caused by the occluded artery;
5. Uncorrectable bleeding diathesis;
6. Refusing surgery due to personal financial burden;
7. Any other conditions not suitable for operation.

### Clinical data collection

We collected the patient’s gender, age, duration of vascular occlusion, past history (hypertension, diabetes, coronary heart disease history), smoking history, drinking history, body mass index (BMI) and occluded stump morphology (the previous neurological symptoms were defined as ipsilateral transient ischemic attacks or ischemic stroke or amaurosis, we record the duration from the last neurological event to intervention, dividing patients into two categories based on whether it is longer than 6 months). Based on the cerebral angiography images, according to the angle formed by the plane of the stump and the vessel wall of the involved blood vessel, it is classified as follows: “sharp” type (≤45°) and “blunt” type (>45°). If the end presents a flat stump, it is the “flat” type; if there is no obvious stump, it is the “no stump” type.

In addition, we also collected and summarized serum laboratory data from various patients: fasting blood glucose value, neutrophil value, lymphocyte value, neutrophil to lymphocyte ratio, platelet count, fibrinogen platelet ratio, platelet lymphocyte ratio, extremely low-density lipoprotein, low-density lipoprotein, apolipoprotein B, triglycerides, cholesterol, uric acid, creatinine, INR, fibrinogen, D-dimer, and foaming test results.

### Evaluation criteria

In this study, the success of endovascular treatment was defined as residual stenosis rate < 50% and establishing grade 3 antegrade TICI flow, with surgical success as the primary endpoint event. TICI is divided into levels 0, 1, 2a, 2b, and 3 (7). The internal carotid artery (ICA) is classified into seven segments (8), namely C1, C2, C3, C4, C5, C6, and C7. The vertebral artery (VA) is classified into four segments, namely V1, V2, V3, and V4. The failure of endovascular treatment is defined when the occluded blood vessel cannot be recanalized after 30 min of repeated attempts and more than 300 milliliters of contrast agent have been consumed, the surgery ended accordingly.

### Interventional techniques, periprocedural management, and medical treatment

The surgery is performed by experienced neurologists under general anesthesia. The guide catheter was advanced into the cervical VA or ICA as high as allowed by vessel tortuosity. A 0.014-inch microwire was carefully coaxially advanced with a microcatheter (Echelon 10) through the occluded segment. The Synchro microwire (200 cm) was our first choice, other micro wires such as Pilot microwires can be replaced appropriately based on the degree of tortuosity of the vascular pathway. After the 0.014-inch microwire with supporting microcatheter passed through the lesion, microcatheter angiography was performed to confirm that the tip of the microcatheter is in the true lumen of the blood vessel. Then, using exchange technology, place a 300 centimeter microwire into the distal vascular branch of the occluded artery. And use a balloon to dilate the narrow segment (at least 30 s). Properly sized balloon stents and self-expanding stents were used to cover narrow segments. Balloon dilation can be performed if the stent expansion is not sufficient.

Each patient should take aspirin, clopidogrel, or ticagrelor orally before surgery. The main lipid-lowering and plaque stabilizing drugs are atorvastatin and rosuvastatin. At the same time, pay attention to observing adverse drug reactions and maintain them for life. If there is a thrombotic event during surgery, tirofiban antiplatelet aggregation therapy can be added. Kang et al. systematically outlined perioperative protocols and pharmacological strategies for cerebral vascular occlusion recanalization (CVOR) procedures (9), and we also referred to this strategy in practical clinical applications.

### Statistical analysis

All continuous variables were expressed as mean ± SD, and categorical variables in numbers and percentage. Chi square test is used to compare categorical data and logistic regression method is used to compare continuous data. All statistical analyses were 2-tailed, and *p* < 0.05 was accepted as statistically significant. Statistical analysis was conducted using R Programming Language (version 4.3.2). Sensitivity and specificity were measured using the Jordan index. To evaluate the sensitivity and specificity of the diagnostic method used in this study, Receiver Operating Characteristic (ROC) curve analysis was conducted. The area under the ROC curve (AUC) was also calculated by R Programming Language. An AUC of 0.5 indicates that the diagnostic test is no better than random chance, while an AUC of 1.0 represents a perfect diagnostic test. The optimal cut - off point for the diagnostic test was determined by maximizing the Youden’s index (sensitivity + specificity − 1). At this cut - off point, the balance between sensitivity and specificity was considered the most favorable for accurate diagnosis.

## Results

A total of 103 consecutive CCAO recanalization attempts were performed from February 2021 to October 2024 in 103 patients (78 men; age 61.1 ± 11.1 years; range: 32 to 81 years) with overall technical success rate 68.9%. Many patients had chronic comorbidities such as hypertension (78, 75.7%), diabetes mellitus (32, 31.10%), and cardiac disease (12, 11.7%). In addition, 38 (36.9%) had a history of smoking, and 23 (22.3%) had a history of drinking. The baseline clinical variables are summarized in Table 1.

**Table 1:** Baseline characteristics of the studied patient population as stratified by failed or successful recanalization.

Characteristics		Failed (*N* = 28)	Successful (*N* = 62)	Chi square value or odds ratio (95% CI)	*p*-value
Gender	Female	7 (25%)	17 (27.4%)	0	1
	Male	21 (75%)	45 (72.6%)		
History of hypertension	No	6 (21.4%)	15 (24.2%)	0	0.9857
	Yes	22 (78.6%)	47 (75.8%)		
History of diabetes	No	17 (60.7%)	46 (74.2%)	1.0887	0.2968
	Yes	11 (39.3%)	16 (25.8%)		
History of coronary heart disease	No	25 (89.3%)	54 (87.1%)	0	1
	Yes	3 (10.7%)	8 (12.9%)		
Smoking history	No	12 (42.9%)	48 (77.4%)	8.8717	0.002896**
	Yes	16 (57.1%)	14 (22.6%)		
History of drinking	No	22 (78.6%)	49 (79%)	0	1
	Yes	6 (21.4%)	13 (21%)		
Stump morphology	Sharp	7 (25%)	44 (71%)	29.827	<0.001***
	Blunt	4 (14.3%)	10 (16.1%)		
	Flat	9 (32.1%)	0 (0%)		
	No stump	8 (28.6%)	8 (12.9%)		
Foaming test	No	22 (78.6%)	42 (67.7%)	0.63711	0.4248
	Yes	6 (21.4%)	20 (32.3%)		
Duration from last neurologic event (longer than 6 months or not)	No	7 (25%)	39 (62.9%)	9.6251	0.001919**
	Yes	21 (75%)	23 (37.1%)		
Neutrophil-to-lymphocyte ratio (NLR)	<2.5	9 (32.1%)	36 (58.1%)	4.1993	0.04044 *
	≥ 2.5	19 (67.9%)	26 (41.9%)		
Age	<60	1 (3.6%)	37 (59.7%)	22.643	<0.001***
	≥ 60	27 (96.4%)	25 (40.3%)		
Neutrophil lymphocytes	<3	0 (0%)	9 (14.5%)	3.0472	0.08087
	≥ 3	28 (100%)	53 (85.5%)		
Glucose		6.0 ± 2.6	5.9 ± 1.7	0.97 (0.78–1.21)	0.796
Lymphocytes, 10^9/L		1.9 ± 0.8	1.9 ± 0.8	1.01 (0.56–1.82)	0.978
Platelets, 10^9/L		254.5 ± 67.7	259.6 ± 65.6	1.00 (0.99–1.01)	0.735
Platelet lymphocyte ratio (PLR)		149.4 ± 61.8	153.9 ± 73.4	1.00 (0.99–1.01)	0.774
Fibrinogen platelet ratio (FPR)		0.0 ± 0.0	0.0 ± 0.0	0.00 (0.00–165.25)	0.279
Very low density lipoprotein, mmol/L		0.4 ± 0.3	0.5 ± 0.3	1.21 (0.22–6.66)	0.830
Low density lipoprotein, mmol/L		2.3 ± 0.7	2.4 ± 0.8	1.15 (0.62–2.13)	0.654
Apolipoprotein B, g/L		0.7 ± 0.2	0.8 ± 0.3	3.26 (0.48–22.19)	0.228
Triglycerides (TG), mmol/L		1.4 ± 0.7	1.5 ± 0.8	1.20 (0.65–2.21)	0.560
Cholesterol (TC), mmol/L		3.7 ± 1.0	3.9 ± 1.1	1.22 (0.77–1.91)	0.398
Uric acid (UA), μmol/L		292.3 ± 79.9	303.9 ± 76.9	1.00 (1.00–1)	0.508
Cr, μmol/L		74.7 ± 22.8	66.2 ± 15.1	0.97 (0.95–1.00)	0.052
Fibrinogen (Fg), g/L		3.2 ± 1.2	3.1 ± 0.9	0.83 (0.54–1.28)	0.401
INR		0.9 ± 0.1	0.9 ± 0.1	0.42 (0.00–399.12)	0.805
IBM		34.1 ± 37.7	26.2 ± 3.5	0.94 (0.83–1.06)	0.318
D-dimer FEU, mg/L		0.5 ± 0.5	0.5 ± 0.7	1.09 (0.52–2.26)	0.818

The value is the mean ± standard deviation or quantity (percentage), represents *p* < 0.1, * represents *p* < 0.05, ** represents *p* < 0.01, *** represents *p* < 0.001.

The rate of overall intraoperative complication was 10.7% (11/103).1 patient had occlusion of ophthalmic artery, 2 patient had a slight subarachnoid hemorrhage followed by microwire perforation, 1 patient had a slight intraparenchymal hemorrhage, 1 patient died of massive recanalization hemorrhage 2 day after the procedure, 2 patients had distal embolization accompanied by mild symptoms of limb hemiplegia. Four patients had reocclusion of instent thrombosis with progression of symptoms of hemiplegia in limbs. No patient dies in the failed recanalization group (Table 2).

**Table 2:** Intraoperative complication.

Intraoperative complication	N
Slight subarachnoid hemorrhage	2
Slight intraparenchymal hemorrhage	1
massive recanalization hemorrhage	1
Distal embolization	2
Reocclusion of instent thrombosis	4
Occlusion of ophthalmic artery	1

**Figure 1:**
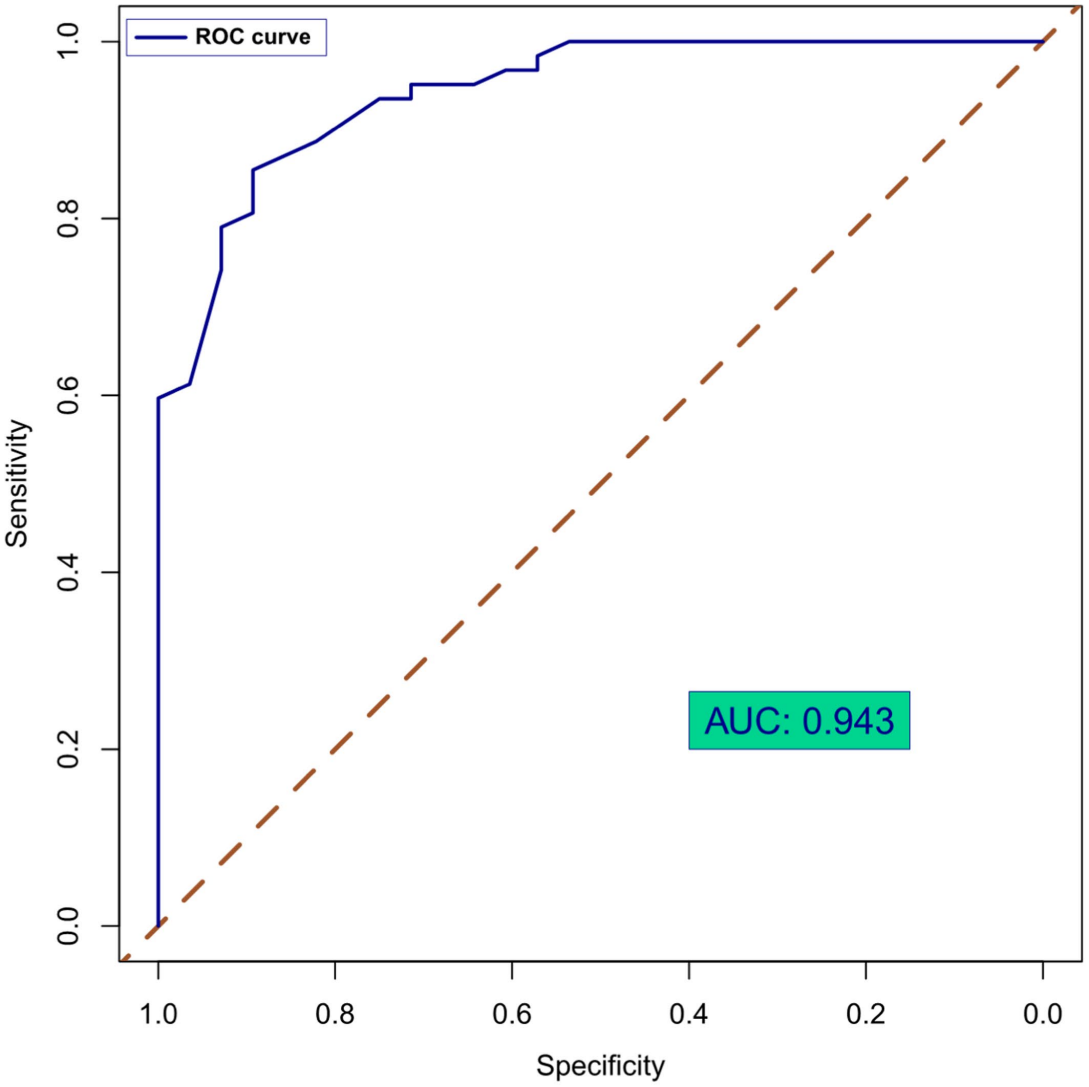
ROC curves of CAO score in predicting technical success.

Table 3 showed the score for predicting surgical success, as well as the success rates of different total score points. The success rate of surgery increases as the score decreases. The c-index on the basis of area under the curve for this scoring system in predicting technical success was 0.943 (95%CI: 0.8905–0.9862; *P* < 0.001), with a sensitivity of 71.28% and a specificity of 75.26 (Figure 1). This indicates that the effectiveness of the model score in predicting patient surgical is commendable.

**Table 3:** Score for predicting surgical success.

	Status	Coefficient	Score
Stump morphology	Sharp	−1.03	0
	Blunt		1
	Flat		2
	No stump		3
Smoking history	No	−2.24	0
	Yes		1
Duration from last neurologic event (longer than 6 months or not)	No	−1.47	0
	Yes		1
Age	<60	−4.77	0
	≥ 60		1
Neutrophil-to-lymphocyte ratio (NLR)	<2.5	−1.98	0
	≥ 2.5		1
Total points		Success rate (%)	
≤6		100	
6 ~ 10		66.7	
≥ 10		11.1	

**Figure 2:**
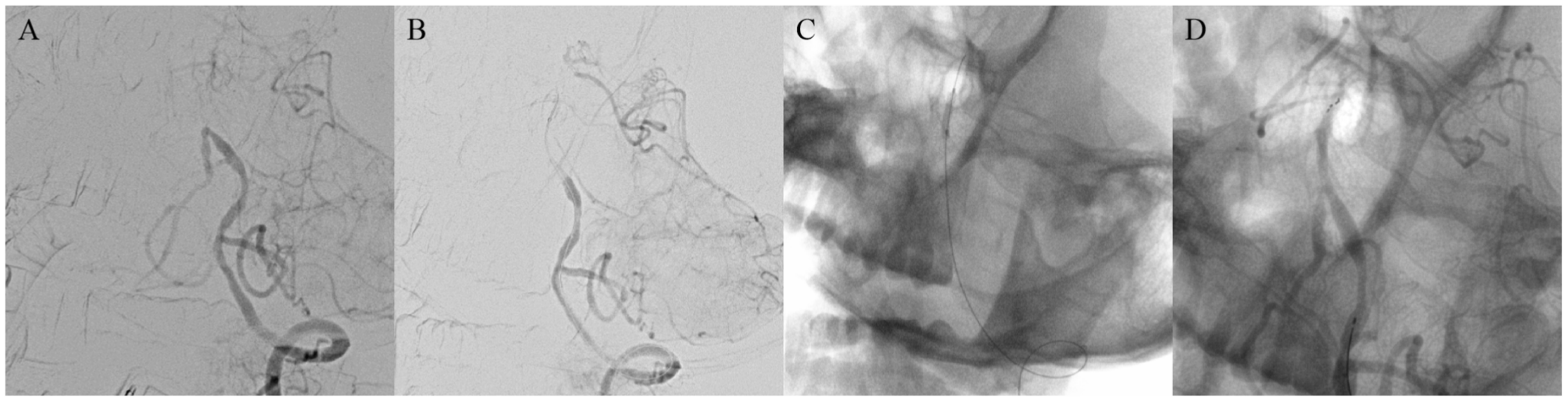
A 42-year-old man with lower basilar artery occlusion. Digital subtraction angiography confirmed occlusion of the basilar artery **(A)**. Carefully probe the blocked section with a micro guide wire and confirm the true lumen of the blood vessel **(B)**. Using balloon catheters to dilate narrow segments of the basilar artery **(C)**. Successful recanalization of basilar artery occlusion, angiography shows good imaging of the basilar artery and bilateral posterior cerebral arteries, with normal blood flow velocity **(D)**.

**Figure 3:**
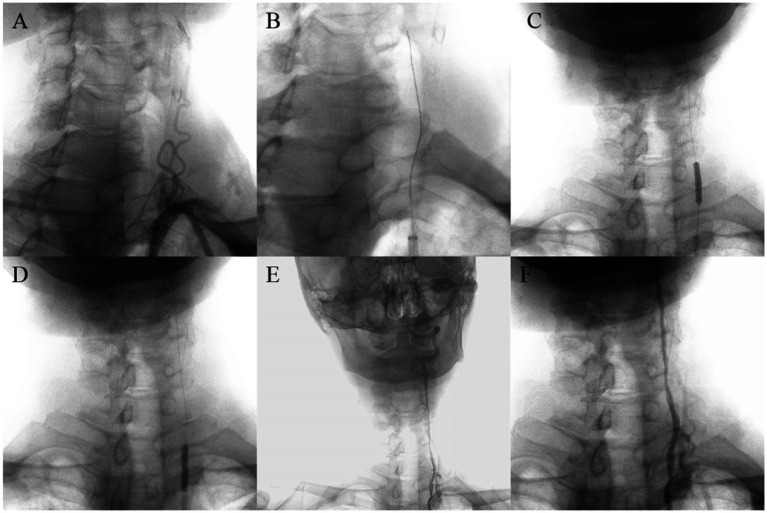
A 69-year-old man with left vertebral artery VI segment occlusion. Digital subtraction angiography confirmed occlusion of the left vertebral artery V1 segment without obvious stumps **(A)**. Carefully probe the blocked section with loach guide wire **(B)**. Using balloon catheters to dilate narrow segments of vertebral artery from far to near **(C,D)**. Successful recanalization with good antegrade perfusion was achieved **(E,F)**.

**Figure 4:**
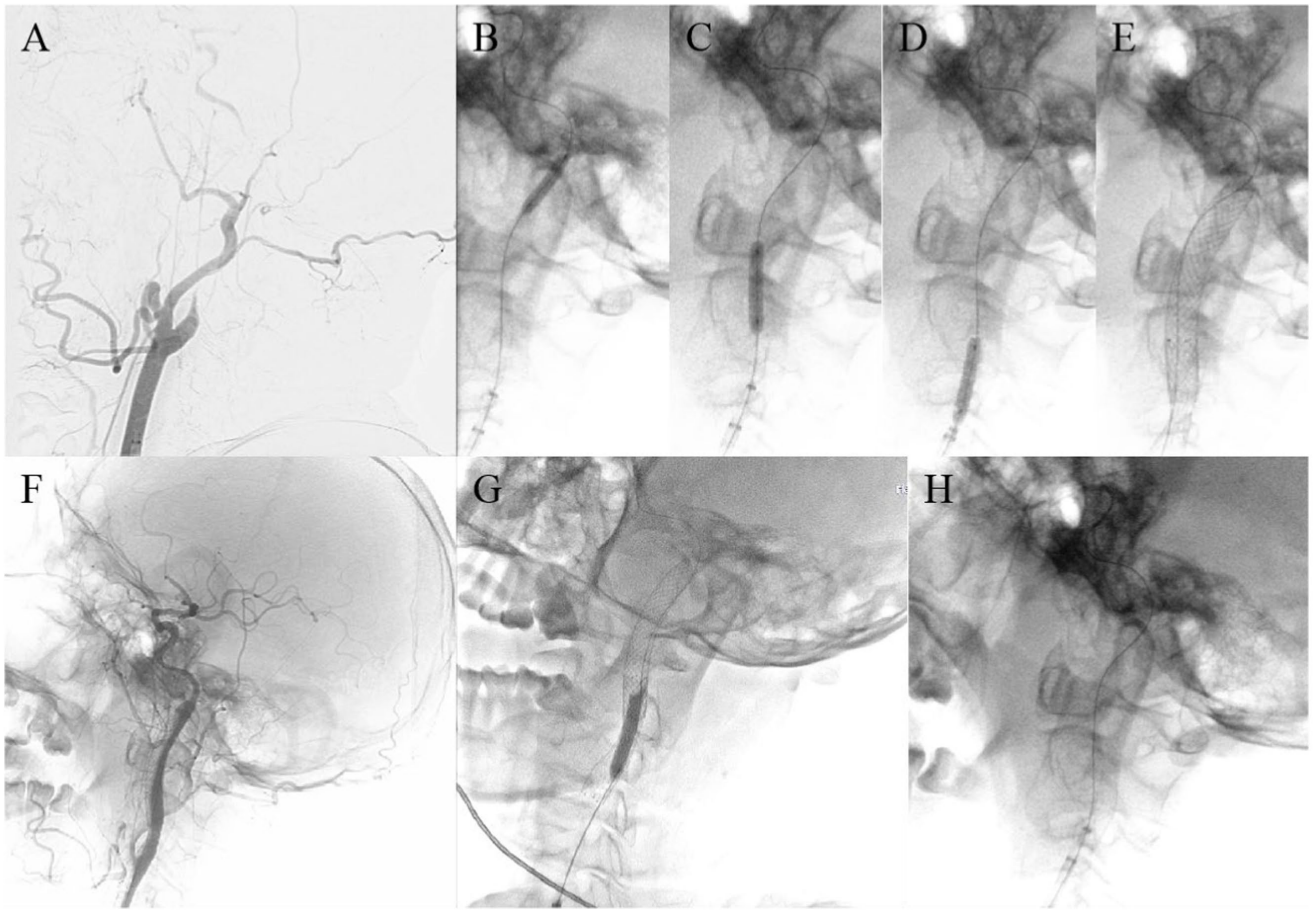
A 64-year-old man with left internal carotid artery C1 segment occlusion. Digital subtraction angiography confirmed occlusion of the left ICA **(A)**. Carefully probe the blocked section with a micro guide wire and confirm the true lumen of the blood vessel **(B)**. Using balloon catheters to dilate narrow segments of the internal carotid artery from far to near in sequence **(C–E)**. Release carotid artery stent **(F)**. The shape of the proximal attachment of the carotid stent for releasing carotid artery stents is slightly poor, and the morphology of arterial stent after release is perfect after redilation with a balloon **(G)**. Successful recanalization of carotid artery occlusion, good imaging of the entire carotid artery, and normal blood flow velocity **(H)**.

**Figure 5:**
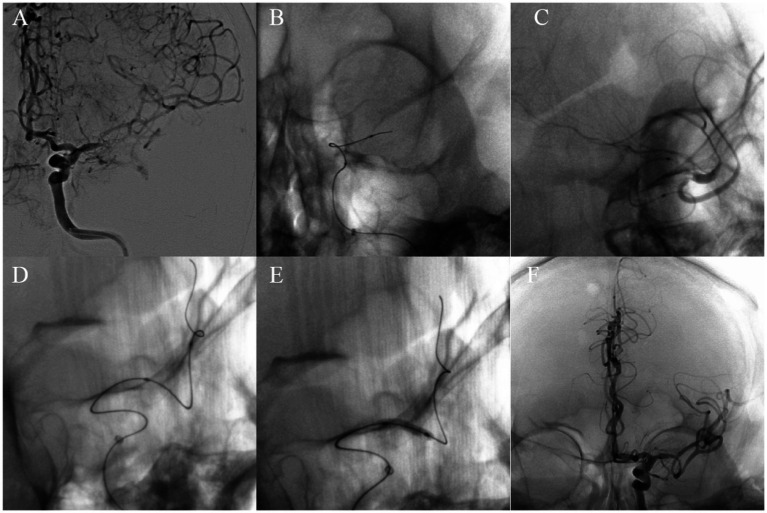
A 65-year-old man with left middle cerebral artery M1 segment occlusion. Digital subtraction angiography confirmed occlusion of the left MCA **(A)**. Carefully probe the blocked section with a micro guide wire and confirm the true lumen of the blood vessel **(B,C)**. Carefully placed the microwire beyond the M2 segment of the middle cerebral artery **(D)**. Balloon dilation of the stenotic segment of the left middle cerebral arterye **(E)**. Successful recanalization with good antegrade perfusion was achieved **(F)**.

According to the type of occlusion site (anterior or posterior circulation, as well as intracranial or extracranial segments), four surgical imaging data of example cases were showed separately as follows (Figures 2–5), including several key steps during surgical process. At the same time we provide corresponding specific descriptions in the diagram.

## Discussion

In terms of the treatment of CCAO, several previous studies have shown that the efficacy of surgical intervention is not very precise, and milestone EC-IC Bypass trial and Carotid Occlusion Surgery Study have not demonstrated any differences in fatal and non-fatal stroke between symptomatic CCAO patients in the surgical and medical groups within 2–5 years (10–12). And due to intolerance to hemodynamic fluctuations during surgery, the perioperative complications in the surgical group are relatively high (13). However, chronic CAO is associated with an annual incidence rate of stroke of 6–20%, despite aggressive medical treatment (14, 15). Intravascular recanalization of CCAO may provide the same recanalization advantage and have a higher safety advantage compared to surgical procedures, which can significantly reduce hemodynamic damage. The successful recanalization of postoperative CCAO patients can improve their cognitive function and quality of life (16–18). Unfortunately, endovascular treatment (ET) technology requires a high level of skill and experience from the surgeon, with potential complications including bleeding, pseudoaneurysm, and carotid cavernous sinus fistula (19), and the success or failure of the surgery is often difficult to predict. Therefore, preoperative evaluation of the system is crucial for identifying patients and lesion features with high success rates, which can help improve the success rate of surgery.

Up to now, experts have developed various predictive assessment scales through statistical analysis, but most of them are only targeted at patients with anterior circulation occlusion. Currently, there is a lack of predictive assessment scales suitable for patients with cerebral vascular occlusion that encompass both the anterior and posterior circulation. Especially there is relatively little research on posterior circulation occlusion.

Jin et al. proposed the application of The HRVWI score system to predict and analyze the successful recanalization of chronic internal carotid artery occlusion (CICAO) (20). Chen et al. developed a scoring system to predict the success rate of intravascular recanalization in CICAO. The predictive factors include absence of perforated stumps, reconstruction of the distal carotid artery in the communicating or ocular segment, and absence of neurological events. However, there is a certain degree of heterogeneity and bias in their research (21). On this basis, Zhou et al. improved the above scoring system and concluded that a residual stump, low levels of the digital ICA occlusion segment, and a short radiological occlusion time were identified as positive predictors of technical success. However, this prediction system is still only applicable to the carotid circulation system (22).

There is relatively little research on the opening of vertebrobasilar artery occlusion, and some scholars have discussed and analyzed it. Gao et al. demonstrated the feasibility and safety of internal veterinary art (ICVA) reanalysis (23). Zhao et al.’s study suggestions that endovascular regression for gut to chronic symptomatic astrological basic art occlusion (BAO) appear to be feasible in selected patients (24). Cai et al.’s research focuses on the treatment of chronic ventricular basic art occlusion in the starting segment (25).

Our study combines cases of the carotid artery system and the vertebrobasilar artery system for analysis, including patient baseline data, imaging characteristics, and laboratory examination data. The final prediction system is applicable to all types of CCAO cases. The final results showed that stump morphology, smoking history, duration from last neurological event (longer than 6 months or not), age, smoking history and neutral to lymphocyte ratio (NLR) were independent risk factors affecting recanalization success. And in this scoring system, we can predict the success rate of re communication based on the score.

It should be noted that there are many peculiarities in cases of vertebrobasilar artery system occlusion. For example, in patients with chronic occlusion of some vertebral arteries with no stump, we can use parallel compensatory vessels (such as the ascending carotid artery) for reverse patency, thereby ensuring the smooth completion of the surgery. However, there is no doubt that no stump is a negative impact factor on the success of the launch. This is also a limitation of this study. Supplementary Figure 1 shows one of the cases of reverse opening of the vertebral artery that we completed.

A study has found that factors affecting bleeding after mechanical thrombectomy for acute anterior circulation occlusion include NHISS score at admission, stroke history, neutrophils, lymphocytes, NLR, PLR, and FPE (26). More and more scholars are becoming enthusiastic about analyzing preoperative laboratory test data of patients in order to explore the influencing factors for predicting surgical outcomes. After occlusion of large blood vessels in the anterior circulation, ischemic brain tissue is stimulated to release various chemokines and cytokines, leading to the entry of white blood cells in the peripheral circulation into the ischemic site. Neutrophils are one of the invading cells and are correlated with the severity of the disease (27–30). Research has found that the accumulation of neutrophils in ischemic areas can exacerbate damage to brain tissue through the release of inflammatory mediators.

Meanwhile, the study also pointed out that severe damage to the blood–brain barrier increases the probability of postoperative bleeding. The interaction between neutrophils and platelets can trigger the formation of new blood clots and exacerbate vascular blockage. Neutrophils have the ability to adhere and aggregate on endothelial cells in ischemic tissue, hindering blood flow (31, 32). These research findings suggest a certain association between neutrophils and postoperative complications. In 2021, studies have indicated the impact of NLR on cerebral artery occlusion recanalization and elucidated its possible pathological mechanisms. When the NLR level is high, it indicates that the patient is likely to have a high neutrophil count or a low lymphocyte count. This situation undoubtedly increases the thrombus burden on occluded blood vessels, thereby increasing the difficulty of thrombectomy device operation and reducing the success rate of cerebral artery occlusion recanalization (33, 34). In our study, it was also confirmed that there is a correlation between NLR and the success rate of occlusive recanalization, and it can serve as a predictive factor, which helps us make relatively more accurate preoperative judgments.

Ter Schiphorst et al. pointed out that the presence of large vessel occlusions (LVO) could represent a “red flag” of PFO causality in stroke of undetermined etiology (35). Therefore, we attempted to explore whether there is a correlation between PFO and chronic occlusion and reperfusion of cerebral arteries. We conducted preoperative foam test screening for each patient, and the final results confirmed that there was no significant correlation between the two. The presence of PFO cannot predict surgical outcomes.

### Study limitations

Although our scoring system is aimed at CCAO patients with different occlusions, the number of cases is still relatively small, especially in cases of vertebrobasilar artery occlusion. This may weaken the predictive ability of the scoring system. We still need to continue expanding case data. More prospective patients are needed in the future to validate current research results. On the other hand, for patients with occlusion of vertebral artery V1 segment, we can attempt reverse recanalization through the ascending carotid artery. Therefore, when predicting the surgical outcome of this type of patient, our application of a scoring system needs to take into account the patient’s arterial collateral compensation situation. In this study, the total number of cases with complications was 11. Considering that a relatively large data bias might occur if a subgroup analysis of complications was carried out, we decided to conduct statistical analysis after further accumulation of the sample size, so as to further enhance the clinical relevance of the research results.

## Conclusion

In summary, CCAO is undoubtedly an important risk factor for ischemic stroke. Our study found that the morphology of occluded stumps, duration from last neurologic event (longer than 6 months or not), age, smoking history and NLR were independent predictors for successful recanalization. The scoring system we have established has high sensitivity and specificity in predicting successful reperfusion, and has a wide range of applications. However, further research is needed to verify its clinical practicality.

## Data Availability

The original contributions presented in the study are included in the article/Supplementary material, further inquiries can be directed to the corresponding authors.
