# Supplementary figures and images for: Endovascular recanalization of symptomatic chronic cerebral artery occlusion: predictors for successful recanalization and perioperative complications
Source: Front Neurol. 2025 Apr 11;16:1453841. doi: 10.3389/fneur.2025.1453841 (PMC12021629; doi:10.3389/fneur.2025.1453841)

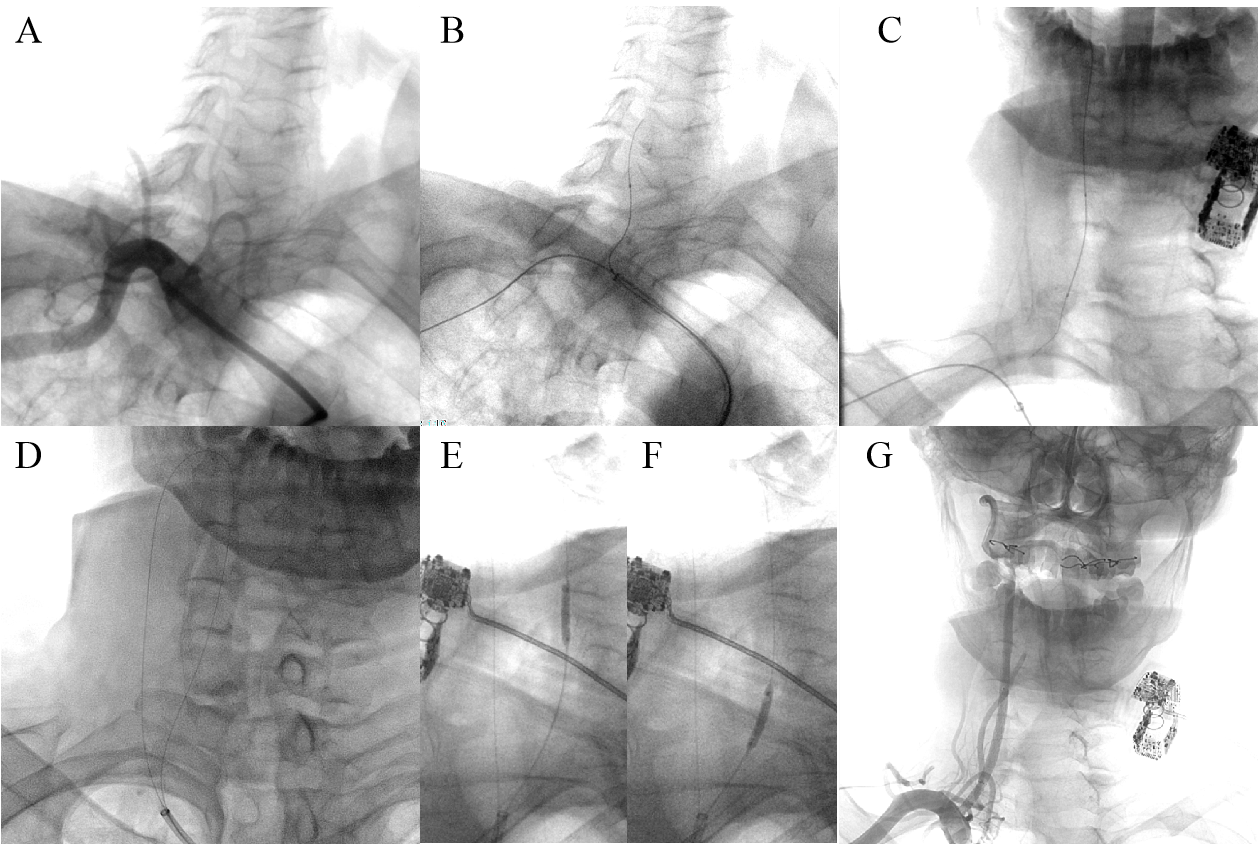

Supplement: Supplementary file 1 [file Image_1.tif]

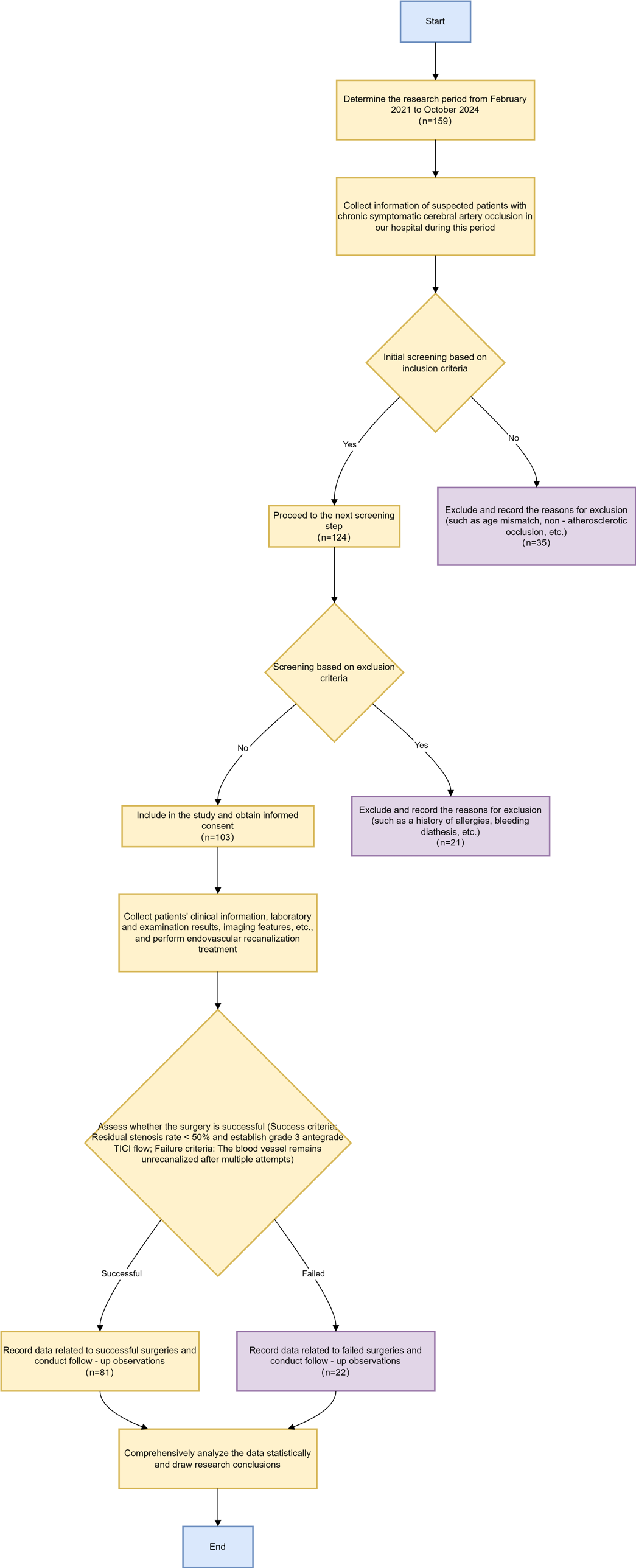

Supplement: Supplementary file 2 [file Image_2.png]
